# Prevalence of needle-stick injury in Iranian nurses: an updated systematic review and meta-analysis of observational studies

**DOI:** 10.1186/s12912-024-01914-z

**Published:** 2024-04-24

**Authors:** Aram Halimi, Reza Ghanei Gheshlagh, Masoumeh Ansari, Seyedeh Soma Zakariaee, Mitra Zandi

**Affiliations:** 1 Research Center for Social Determinants of Health, Research Institute for Endocrine Sciences, Shahid Beheshti University of Medical Sciences, Tehran, Iran; 2 Department of Epidemiology, School of Public Health and Safety, Shahid Beheshti University of Medical Sciences, Tehran, Iran; 3 Clinical Care Research Center, Research Institute for Health Development, Kurdistan University of Medical Sciences, Sanandaj, Iran; 4 Department of Medical Librarianship and Information Sciences, School of Health Management and Information Sciences, Iran University of Medical Sciences, Tehran, Iran; 5 Student Research Committee, School of Health Management and Information Sciences Branch, Iran University of Medical Sciences, Tehran, Iran; 6 Department of Midwifery, Faculty of Nursing and Midwifery, Kurdistan University of Medical Sciences, Sanandaj, Iran; 7 Department of Medical-Surgical Nursing, Faculty of Nursing & Midwifery, Shahid Beheshti University of Medical Sciences, Tehran, Iran

**Keywords:** Needle stick injury, Nurse, Prevalence

## Abstract

**Background:**

The aim of this study was to conduct a systematic review and meta-analysis to estimate the prevalence of needle-stick injury among Iranian nurses.

**Methods:**

We conducted a systematic review and meta-analysis to estimate the prevalence of needle-stick injury among Iranian nurses. A comprehensive search of Web of Science, PubMed, Scopus, Scientific Information Database, and MagIran was performed, yielding 29 observational articles comprising 8842 nurses. The studies ranged from 2006 to 2023, with sample sizes varying from 68 to 1555 individuals. Methodological quality was assessed using the Strengthening the Reporting of Observational Studies in Epidemiology checklist. The pooled prevalence was calculated using the random-effects model, and subgroup analyses were conducted based on hospital type and gender. The data was analyzed using Stata software version 16.

**Results:**

The pooled prevalence of needle-stick injury among Iranian nurses was found to be 46% (95% Confidence Interval [CI]: 39-53%). Subgroup analysis revealed significant difference in prevalence between teaching hospitals (47%; 95% CI: 39-54%) and military hospitals (38%; 95% CI: 31.1-44%). The prevalence of NSI in region 1 (Tehran and surrounding provinces) and other regions was 45.1% (95% CI: 37-54%) and 49.17% (95% CI: 36.5-61.7%). Gender-based analysis showed higher prevalence in women (58%; 95% CI: 44-71%) compared to men (55%; 95% CI: 43-66%).

**Conclusion:**

Needle stick injuries has a high prevalence among Iranian nurses, especially nurses working in teaching hospitals. Therefore, it seems necessary to use interventions to reduce it.

## Introduction

Needle-stick injury (NSI) poses a significant occupational hazard for healthcare workers, particularly nurses, involving the unintended penetration of a needle into the skin [1]. While NSI is preventable, its occurrence exposes individuals to over 20 different blood-borne pathogens [2]. Annually, more than two million healthcare workers, facing potential exposure to blood-borne infectious diseases such as hepatitis B and C, and human immunodeficiency virus (HIV) [3] undergo NSIs. Additionally, NSI has been implicated in the transmission of other infections, including diphtheria, herpes, malaria, and syphilis [4]. Beyond the physical risks, victims may encounter psychiatric complications such as post-traumatic stress disorder and psychological distress [5]. 

The repercussions of NSI extend beyond the individual health worker, impacting their well-being and creating fear and anxiety that can diminish the quality of life and care they provide. The economic, clinical, and human burden of these injuries necessitates careful consideration by healthcare managers [6, 7]. In resource-limited healthcare settings like Iran, the economic burden of NSI is particularly pronounced [7, 8]. Contrary to expectations, the costs associated with NSIs, both direct and indirect, are considerably high, while the costs of prevention remain comparatively low [9, 10]. In Japan, for instance, the estimated costs of NSIs are substantial, averaging $577 per injury and escalating to $1333, $936, and $2743 if infected with hepatitis B and C, and HIV, respectively [11]. Despite the potential benefits of rapid and accurate NSI reporting for treatment, more than half of Iranian nurses refrain from reporting their injuries due to various reasons [12]. 

A recent study on the prevalence of NSI in Iranian hospitals indicated a noteworthy prevalence rate of 42.5% among healthcare workers [13]. Given the occupational nature of nursing, nurses appear to be more susceptible to NSI than their counterparts in other healthcare professions [14, 15]. Notably, various studies in Iran have reported divergent prevalence rates of NSI among nurses, ranging from 26–81% [16, 17]. To address this issue, it is crucial to establish a clear understanding of the current prevalence of NSI among Iranian nurses. Consequently, this systematic review and meta-analysis aim to provide a comprehensive estimate of the overall prevalence of NSI in this specific population.

## Methods

This study was a systematic review and meta-analysis based on the preferred reporting items for systematic reviews and meta-analyses (PRISMA) guidelines but its protocol was not recorded in the international prospective register of systematic reviews (PROSPERO).

### Search strategy

In this study, studies that were published in English and Persian from 2006 till July 2023 were included. Studies that the prevalence of NSI among Iranian nurses without a time limit was investigated. For this purpose, databases, Web of Science, PubMed and Scopus, Scientific Information Database (SID), MagIran were searched with the following keywords: “needlestick injuries” OR “needle? stick Injur*” OR “needle? stick*” OR “sharps injur*” AND “health Personnel” OR “health personnel” OR “health care provider*” OR “healthcare worker*” OR “health care professional*” OR “nurse AND “Iran” OR “Iran*” OR “Islamic Republic of Iran”. The sources of the collected articles were also reviewed for access to other articles to ensure that all potentially relevant articles were collected.

### Selection of studies and data extraction

We reviewed all studies published in Persian and English that examined the prevalence of NSI in Iranian nurses. Inclusion criteria were: observational studies, access to the full text of the article, performing a study on nurses, and the existence of essential information in the article. In some studies, the prevalence of NSI was observed in all healthcare workers. Those articles were reviewed, and if the prevalence was reported separately in nurses, we would record it, and if the data were presented separately, we would calculate the prevalence in nurses. Studies performed on other healthcare workers or students and studies that did not report an outbreak, or whose full text was not available, were excluded. According to the inclusion and exclusion criteria, the two researchers independently reviewed the titles and abstracts of the articles and separated the relevant items (first author, year of publication, sample size, mean age of nurses, and place), and recorded the information required for analysis in a pre-prepared form. Disagreements between the two reviewers were resolved by discussion.

### Quality assessment

The included studies underwent an evaluation of their methodological quality and risk of bias using The Joanna Briggs Institute Critical Appraisal tool designed for cross-sectional studies. This checklist focuses on evaluating the quality of cross-sectional studies, covering 9 domains. An overall score exceeding 7 indicates high quality, while a score between 4 and 6 suggests medium quality, and a score below 3 indicates poor quality [17]. 

### Statistical analysis

Given that the prevalence of NSI had a normal distribution, we calculated the variance of each study through the variance of the normal distribution, as var $$(\bar x) = {\raise0.7ex\hbox{${{\sigma ^2}}$} \!\mathord{\left/{\vphantom {{{\sigma ^2}} n}}\right.\kern-\nulldelimiterspace}\!\lower0.7ex\hbox{$n$}}$$. The weight of each study was proportional to its inverse variance. I [2] index and Cochran’s Q test were used to evaluate the heterogeneity of data. If the I [2] index is more than 50%, or the probability value of the Q test is significant, the random-effects model is used, otherwise, the fixed effects model is used. This index can estimate the observed differences between studies due to heterogeneity. A value of 0% indicates no heterogeneity and a value of 100% indicates the highest level of heterogeneity [18]. To calculate the prevalence of NSI by hospital type and gender, subgroup analysis and to investigate the relationship between the prevalence of NSI and the year of publication and sample size, meta-regression was used. All analyzes were performed with STATA version 16.

## Results

**Fig. 1:**
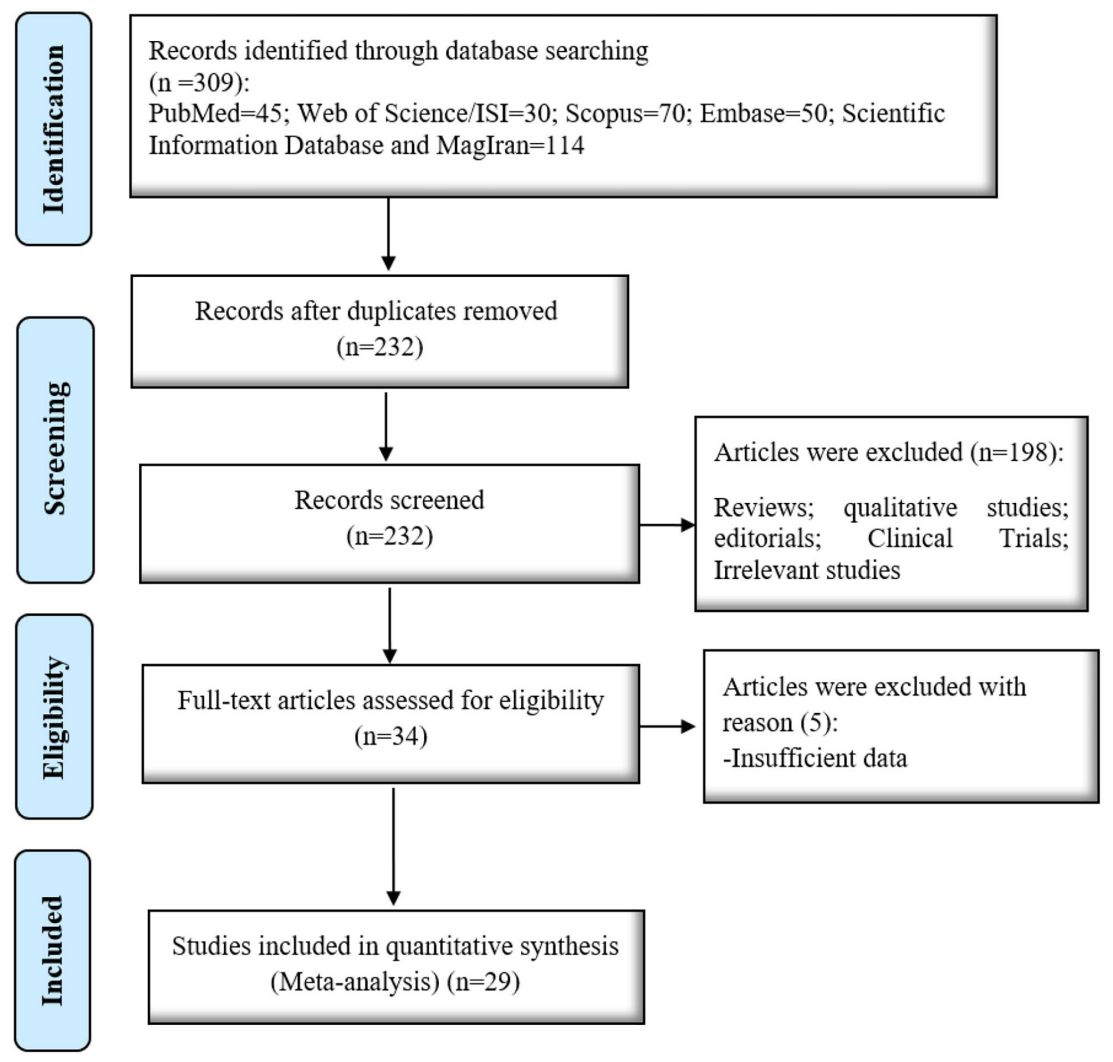
Articles selection process

A total of 29 articles were obtained. Seventy-seven articles were removed due to duplication and 232 articles remained. The two authors independently reviewed the titles and abstracts of the articles, excluded 198 unrelated articles, and reviewed the full text of the remaining 34 articles. Five studies were omitted due to failure to report essential information (Fig. 1).

### Characteristics of included studies

A total of 29 observational articles performed on 8842 nurses were analyzed. The earliest and latest articles were published in 2006 and 2023, respectively. The sample size in included studies ranged from 68 to 1555 people. Three studies were conducted in military hospitals and the rest in teaching hospitals. In terms of methodological quality, all studies had the lowest bias (Table 1).

**Table 1:** Characteristics of the included studies in the meta-analysis of the prevalence of NSI among nurses in Iran, 2023

First author	Year	Place	Sample size	Prevalence (%)	Findings	Quality
Roozbeh [19]	2023	Shiraz	106	19.8	NSI prevalence was significantly higher among those with higher age (*p* = 0.033), work experience > 10 years (*p* = 0.040), and those who graduated earlier (*p* = 0.031). The intravenous injection was the most common procedure leading to NSI, and being in a hurry was the most common cause. The average general health was 3.7 ± 3.2, higher among those not exposed to NSI (*p* = 0.042)	High
Askari Majdabadi [20]	2022	Tehran	200	45.4	The results showed that increasing risk-taking, increasing aggression, decreasing self-confidence, and decreasing emotional intelligence reduced safe behavior and increased the number of needle injuries (*P* < 0.001).	High
Rashidi [21]	2020	Khorramabad	380	53.7	Most nurses were female (79.2%), and in the range of 20 to 30 years (52%). There was a significant correlation between NSI with shift work (*p* = 0.003), work experience (*p* = 0.027), and workplace (*p* = 0.015).	High
Joukar [22]	2018	Rasht	1010	57.4	Most of the participants were female (94%), in the range of 30 to 40 years (39.3%), and had more than 10 years of work experience (40.8%). 42.2% of the samples had experienced NSI more than once. Also, 63.6% of NSI cases occurred in the morning shift.	High
Ramzani [23]	2018	Sari	132	38.1	Most participants were female (60.6%) and married (77%). The main reason for needle sticking in the view of nurses (62.7%) was high workload.	Medium
Ghasemi [24]	2017	Tehran	267	41.2	Most participants were female (52%) and had a bachelor’s degree (98%). The mean age of the samples was 33 years.	Medium
Abareshi [25]	2017	Sabzevar	204	33	Most of the participants were female (65.2%) and half of the participants were under 30 years old. Most of the injured nurses (84.62%) used personal protective equipment during the NSI and most of them (73.15%) had no training in this regard.	Medium
Mehravar [26]	2016	Gorgan	345	81.3	Most participants were female (69.5%). The mean age of participants was 30.9 years. Re-capping has been reported as the most important cause of NSI. A significant relationship has been reported between gender and NSI (*p* = 0.016).	Medium
Jahangiri [27]	2016	Shiraz	168	76	Most of the participants were women (72.6%) and had a bachelor’s degree. There was a significant relationship between the occurrence of NSI with gender (*p* = 0.024), number of working hours per week (*p* = 0.001), and number of shifts per month (*p* = 0.041).	High
Hajivandi [28]	2015	Bushehr	68	55.8	Most NSI (80%) were caused by needles. Nurses considered high workload as the main reason for their injury.	Medium
Mahmoudi [29]	2015	Tehran	100	41	Most participants were female (69%). The mean age and work experience of nurses were 34.7 years and 10.99 years, respectively. Most nurses (63%) reported their injury to their authorities. There was a significant relationship between NSI with age, work experience and education, and workplace (*p* < 0.05).	Medium
Balouchi [30]	2015	Kerman	200	64	Most participants were female (86%) and married (84.5%). In this study, there was no significant relationship between NSI history, age, work experience, and number of shifts per month.	Medium
Mirzaei-Alavijeh [31]	2014	Rafsanjan	70	41.4	More than half of the participants were male (53.4%) and single (58.6). The mean age and work experience of nurses were 29.7 and 6.98 years, respectively. There was a significant relationship between NSI and job stress and gender (*p* < 0.05).	Medium
Ghanei Gheshlagh [32]	2014	Saghez	120	44.2	More than half of the participants (55.8%) were women. The mean age and work experience of nurses were 33.4 years and 9.8 years, respectively. Most of the samples (76.7%) had a bachelor’s degree or higher. There was a significant relationship between the prevalence of NSI with younger age and low work experience.	Medium
Bijani [33]	2014	Qazvin	246	31.3	The majority of participants (85%) were female. Needle re-capping and the number of repeated shifts were associated with NSI.	Medium
Rezaei [34]	2013	Tehran	514	26	Most participants (94%) were female and (63.43%) were married. A significant relationship was reported between NSI and work experience (*p* < 0.05).	Medium
Tirgar [35]	2012	Babol	333	59.7	Most participants were female (80%) and had less than 10 years of work experience. Their average working hours per week were 54.2 h. The variables of age, work experience, training and needle repositioning had a significant relationship with NSI.	Medium
Ehsani [36]	2012	Tehran	328	45.1	Most participants were female (70.42%) and married (60.36%). The mean age and work experience of nurses were 30.71 years and 9.03 years, respectively. There was a significant relationship between NSI and age and ward.	Medium
Mohammadi [37]	2011	Qazvin	138	38.4	There was a significant relationship between NSI and ward.	High
Bijani [38]	2011	Qazvin	172	32	Most nurses were female (84.9%). The most common cause of NSI was syringes (70.9%) and most injuries occurred during blood sampling (36.4%). There was a significant relationship between NSI and consecutive shifts.	Medium
Rahnavard [39]	2011	Rasht	500	77.2	Most NSIs occurred during night shifts in nurses over 40 years of age. Needle was the most common cause of NSI.	Medium
Khalooei [40]	2010	Kerman	338	33	The mean age and work experience of nurses were 34 years and 11.7 years, respectively. Most injuries (29.6%) occurred in the surgical ward. Most injuries occurred during blood sampling (28.3%) and in the morning shift (37.7%).	Medium
Mohammadi Nejad [41]	2010	Tehran	68	47	Most participants (88.2%) were female, married (52.9%), and had less than 5 years of work experience (64.7%). There was a significant relationship between work experience and NSI (*p* < 0.05).	Medium
Kazemi Galougahi [42]	2010	Tehran	158	57	Most participants (59.5%) were female. Most injuries (24.44%) occurred during injections. Most NSIs were related to the operating room (18.9%).	Medium
Mohammad Nejad [43]	2009	Tehran	218	43.1	The most important cause of injury from the nurses’ point of view was overcrowding. 32% of nurses did not report their injury and the most common reason for non-reporting by nurses was dissatisfaction with follow-up and unfamiliarity with the reporting process.	Medium
Joneidi Jafari [44]	2008	Tehran	613	32.7	More than half (53.2%) of the injured nurses were male. The highest prevalence of NSI (22.9%) was related to the internal ward. The most common cause of NSI (70.6%) was the syringe. Most injuries (24.4%) occurred during blood sampling and in the morning shift (37.8%).	Medium
Azadi [45]	2007	Tehran	111	45	Most nurses were women (73.9%) with less than ten years of work experience (69%). Most injuries (38%) occurred due to recapping. Work experience and type of shift were associated with NSI.	Medium
Askarian [46]	2007	Shiraz	1555	26.3	Most participants (77%) were female. The mean age and work experience of nurses were 31 years and 7 years, respectively. A significant relationship was reported between NSI and work experience and age.	High
Ebrahimi [47]	2006	Shahrud	180	63.3	Most participants (68.3%) were female and had less than ten years of work experience (65%). Most injuries occurred during injections.	Medium

### Prevalence of NSI

**Fig. 2:**
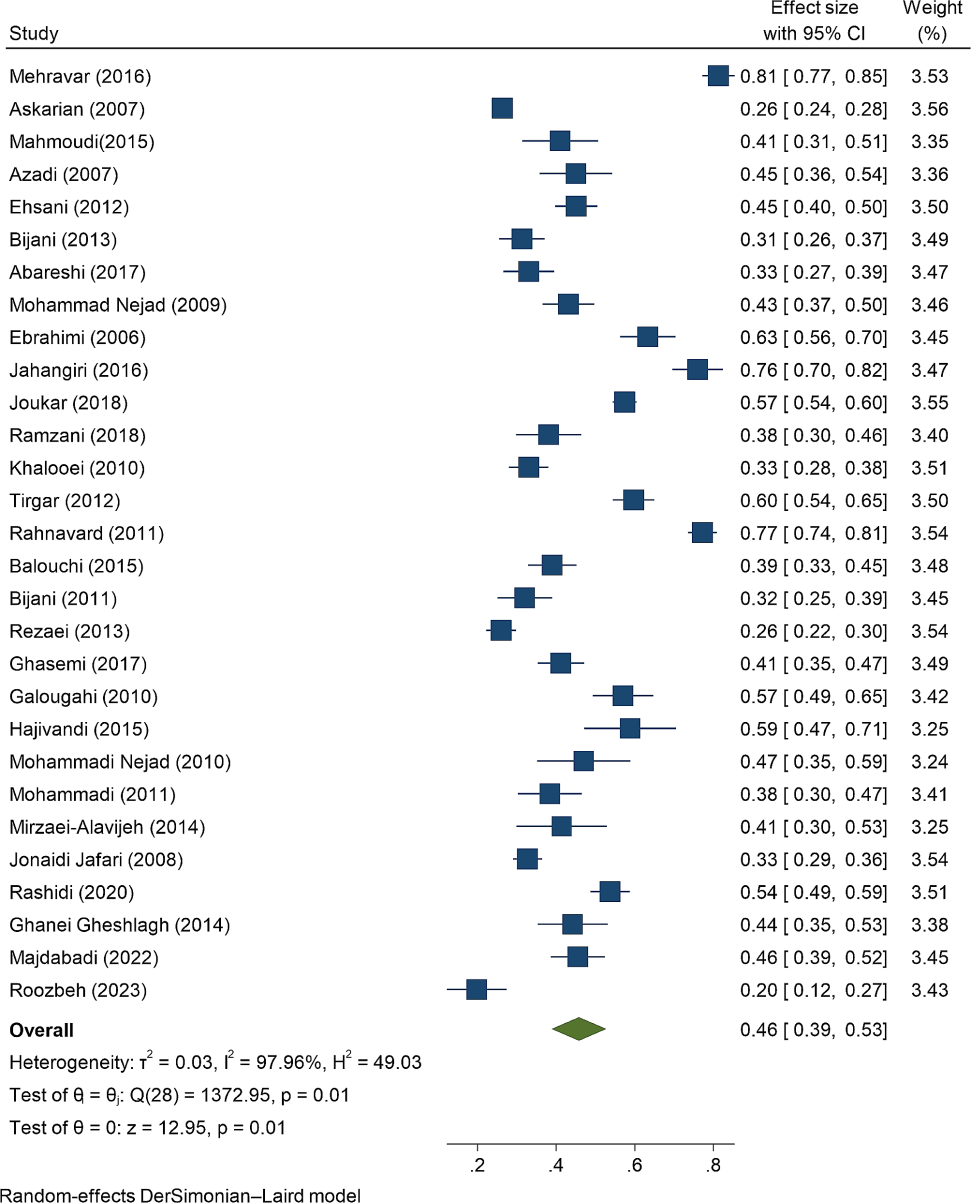
Funnel plots of the pooled prevalence of the NSI among the Iranian nurses

The prevalence of NSI in selected studies ranged from 19.8 to 81.3%. The pooled prevalence of NSI using the random-effects model was 46% (95% Confidence interval [CI]: 39-53%) (Fig. 2). The results of subgroup analysis showed that the prevalence of NSI in teaching hospitals (47%; 95% CI: 39-54%) was significantly higher than in military hospitals (38%; 95% CI: 31.1-44%) (*p* = 0.04, Q = 4.06). Also, the pooled prevalence of NSI in studies conducted in region 1 (Tehran and surrounding provinces) was 45.1% (95% CI: 37-54%) and in other regions was 46.5% (95% CI: 36.5-61.7%) (*p* = 0.437, Q = 0.05) (Table 2).

### Quality assessment

The quality of 29 included articles were high or medium that it was according to the number of the overall score of their quality assessment. Articles were high quality had an overall score exceeding 7 indicates and articles were medium quality had a score between 4 and 6 suggests medium quality. The prevalence of NSI in high (*n* = 7) and medium (*n* = 22) quality studies was 46.1% (95% CI: 30.6-61.6%) and 45.6% (95% CI: 39.6-51.7%), respectively (*p* = 0.956).

**Table 2:** The results of subgroup analysis

Group		Number of studies	Pooled prevalence (95% CI)	I^2^	p
Quality	High	7	46.1% (30.6-61.6%)	98.9%	0.956
	Medium	22	45.6% (39.6-51.7%)	95.3%	
Region	Region 1	17	45.1% (37-54%)	95.2%	0.437
	Other regions	12	49.17% (36.5-61.7%)	98.3%	
Hospital	Teaching	26	47% (39-54%)	97.2%	0.044
	Military	3	38% (31.1-44%)	68.4%	

The prevalence of NSI in men and women was also reported separately in nine studies. Accordingly, the prevalence of NSI in men and women was 55% (95% CI: 43-66%) and 58% (95% CI: 44-71%), respectively. The prevalence of NSI was higher in men in region 1 than in other regions (57.2% vs. 50.8%); but the prevalence of NSI in women in region 1 was lower than in other regions (55% vs. 61.2%). The present meta-analysis was highly heterogeneous due to differences in study quality, study methodology, and sample size between studies (I^2^ = 97.2%, *p* = 0.01). Therefore, we used the random-effects model to adjust the observed variability. The results of meta-regression showed that there was no relationship between the prevalence of NSI and the year of publication of articles and the sample size of articles.

### Publication bias

**Fig. 3:**
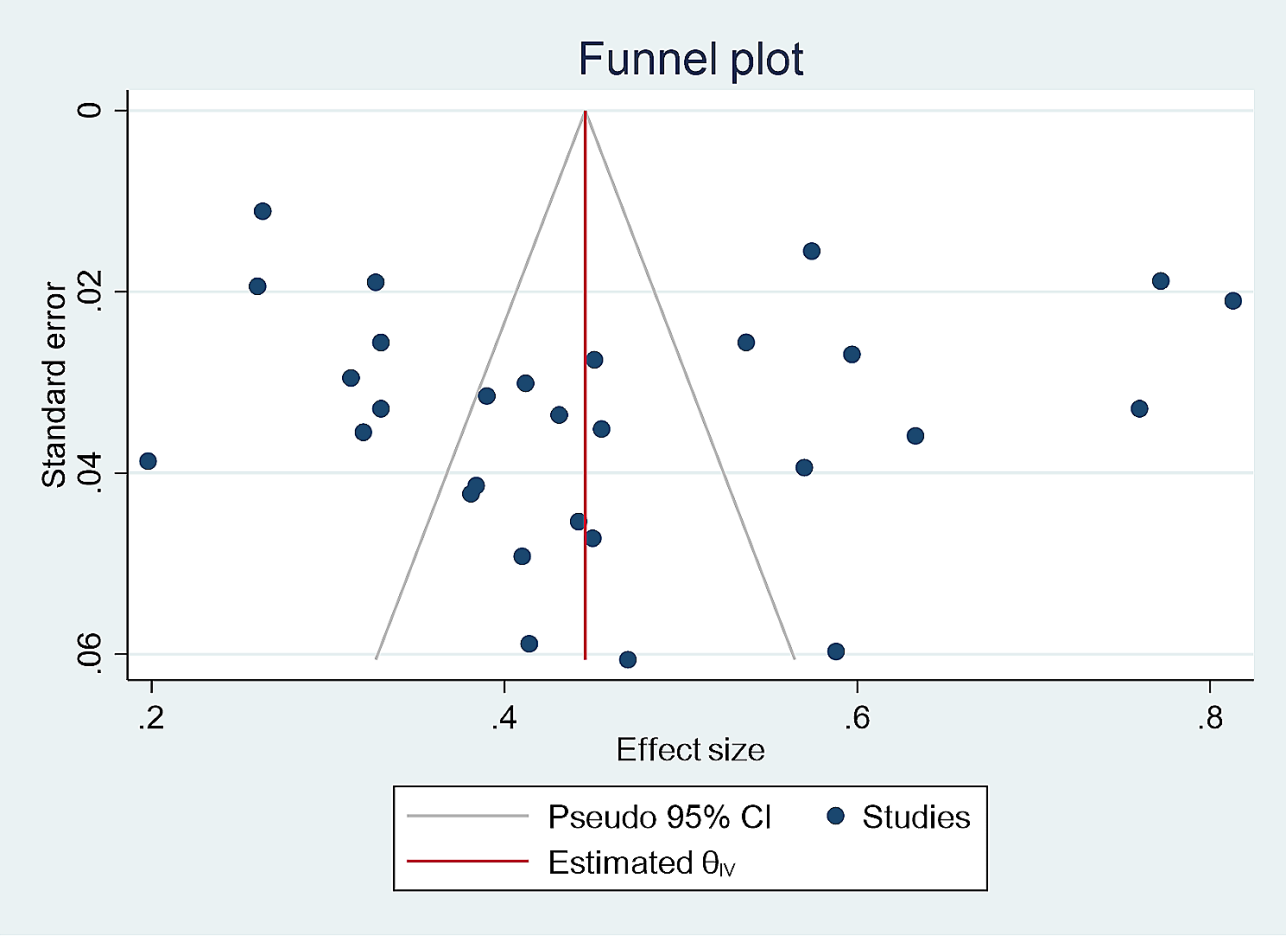
Publication bias

To evaluate the publication bias, we used funnel plots and Egger’s test, in which each point on the funnel plot represents a separated study, and the symmetrical distribution indicates that there is no publication bias. According to Egger’s test, the publication bias was not significant (*p* = 0.833) (Fig. 3).

## Discussion

The results of this study indicate that nearly half of Iranian nurses have experienced NSIs. This prevalence rate is higher than those reported by two separate studies conducted in Turkey and Qatar, which found that 30.1% and 20.9% of nurses, respectively, had experienced NSIs [48, 49]. 

Xu et al. in 2022 conducted a systematic review and meta-analysis study into the prevalence of NSIs among nursing students. The results showed that nursing students reported a notable 35% prevalence of NSIs. Furthermore, regional variations were observed, with Asia exhibiting the highest prevalence at 39.7%, highlighting differences in the incidence of NSIs among nursing students across geographical areas [50]. In another systematic review and meta-analysis study that was done by Dechasa Adare Mengistu et al. in 2021, the global combined prevalence of needle stick injuries among healthcare professionals throughout their career and in the preceding year was 56.2% (95% CI: 47.1, 64.9) and 32.4% (95% CI: 22.0, 44.8), respectively [51]. And a comprehensive systematic review and meta-analysis by Abdelmalik et al. (2023) underscore the global prevalence of NSI among nurses, revealing an overall pooled prevalence of 40.97%. Their study further delineates variations in NSI prevalence based on World Health Organization (WHO) regions, socioeconomic development index (SDI), and the developmental status of countries. In particular, Southeast Asia exhibited the highest prevalence at 49.9%, while the United States of America reported the lowest at 25.1%. Additionally, the study highlights a distinction between developed (30.5%) and developing countries (46.6%), with low-middle SDI countries experiencing the highest NSI prevalence at 48.9% [52]. 

The high prevalence of NSIs in Iran may be due to factors such as the shortage of nursing staff caused by retirement and immigration, which leads to the employment of newly graduated nurses who lack clinical and nursing experience. It is important to note that the high prevalence of NSIs in Iran is not limited to nurses, but also affects other healthcare workers. A meta-analysis by Ghani Gheshlagh et al. (2018) revealed that 44% of nurses and 41% of other healthcare workers had experienced NSIs [13]. These findings show an increase in the prevalence of NSIs compared to previous meta-analyses. Moreover, the prevalence of NSIs is higher among female nurses than male nurses, which may be attributed to the higher levels of occupational stress experienced by women.

The prevalence of NSIs in female nurses was higher than that of male nurses in our study. We attributed this finding to the high level of occupational stress experienced by women. This result is consistent with a systematic review and meta-analysis by Hassanipour et al. (2021), which examined the prevalence of NSIs among healthcare workers and found that it was higher in women than in men [53]. The reason for the high prevalence of NSIs in women may be due to their busyness and responsibility [32]. However, some other studies have reported that the prevalence of NSIs is higher in men than in women [54–56]. These conflicting results indicate that NSIs are very common among nurses, regardless of their gender. Teaching hospitals had a higher prevalence of NSI than military hospitals. According to the Iranian Ministry of Health’s guidelines, nursing graduates are required to work in teaching hospitals for one to two times the length of their studies. The high prevalence of NSI in teaching hospitals could be due to the large number of inexperienced nurses working there.

Considering the lack of relationship between the year of publication of articles and the prevalence of NSIs, it can be inferred that NSIs have not changed significantly from 2006 to 2023. In other words, the general policies regarding the prevention and reduction of NSIs have not been effective in this 17-year period.

### Strengths of the study

In conducting this systematic review and meta-analysis on the prevalence of needlestick injuries (NSI) among Iranian nurses, several strengths contribute to the credibility and reliability of the findings.

1. **Comprehensive Search Strategy**: Our study employed a thorough search strategy encompassing diverse databases and languages, ensuring the inclusion of a wide array of relevant studies on NSI prevalence among Iranian nurses.
2. **Clear Inclusion Criteria**: Well-defined inclusion criteria, including specific study types, language parameters, and a defined timeframe, enhance the transparency of study selection, providing a solid foundation for the research.
3. **Adherence to PRISMA Guidelines**: The adherence to Preferred Reporting Items for Systematic Reviews and Meta-Analyses (PRISMA) guidelines underscores the methodological rigor of our study, reinforcing the systematic and transparent nature of our approach.
4. **Methodological Quality Assessment**: Utilizing the Joanna Briggs Institute Critical Appraisal tool for cross-sectional studies to assess methodological quality and bias adds a layer of robustness to the study, ensuring a critical evaluation of the included articles.
5. **Statistical Rigor**: The use of advanced statistical methods, including subgroup analysis, meta-regression, and consideration of heterogeneity, not only reflects the depth of our analysis but also contributes to the validity of the pooled prevalence estimate.

### Limitations of the study

While our study provides valuable insights into NSI prevalence among Iranian nurses, it is important to acknowledge certain limitations that may influence the interpretation of our findings.

1. **Protocol Registration Absence**: The absence of our study protocol in the international prospective register of systematic reviews (PROSPERO) limits the transparency of our methods, potentially impacting the reproducibility of our study.
2. **Language Bias**: Our study’s inclusion of articles only in English and Persian may introduce language bias, potentially excluding pertinent studies published in other languages.
3. **Heterogeneity Effect**: The identified high heterogeneity in our study, addressed through the use of a random-effects model, underscores the variability between studies, potentially impacting the reliability of the pooled prevalence estimate.

### Overall GRADE assessment

Considering the GRADE [57] approach, the quality of evidence for the study results varies across domains. While the study exhibits strengths in addressing publication bias and heterogeneity, limitations related to study protocol registration, language bias, and regional focus contribute to a moderate overall quality of evidence. The transparency in reporting these considerations enhances the reader’s ability to critically evaluate the reliability and generalizability of the presented results.

### GRADE assessment of study results

### 1. Study limitations

- **Assessment**: The study clearly outlines its inclusion criteria, methodology, and statistical analyses. However, the absence of a registered protocol in PROSPERO and the focus on studies published in English and Persian may introduce limitations.
- **GRADE Evaluation**: Low to moderate quality.

### 2. Inconsistency

- **Assessment**: The study acknowledges high heterogeneity, which is appropriately addressed using a random-effects model. Subgroup analyses were conducted to explore potential sources of variability.
- **GRADE Evaluation**: Moderate quality.

### 3. Indirectness

- **Assessment**: The study primarily focuses on Iranian nurses, potentially limiting generalizability to a global context. The regional and hospital-type variations are considered in subgroup analyses.
- **GRADE Evaluation**: Moderate quality.

### 4. Imprecision

- **Assessment**: The study provides a large sample size and a narrow confidence interval for the pooled prevalence estimate. However, variability in sample sizes across individual studies is noted.
- **GRADE Evaluation**: Moderate to high quality.

### 5. Publication bias

- **Assessment**: Publication bias was assessed using funnel plots and Egger’s test, and the results indicated no significant bias.
- **GRADE Evaluation**: High quality.

### Overall GRADE assessment

Considering the GRADE approach, the quality of evidence for the study results varies across domains. While the study exhibits strengths in addressing publication bias and heterogeneity, limitations related to study protocol registration, language bias, and regional focus contribute to a moderate overall quality of evidence.

## Conclusion

NSI is highly prevalent in Iranian nurses, so that nearly half of them have experienced NSI. The highest prevalence of NSI was related to female nurses and nurses working in teaching hospitals. Considering the high prevalence of NSI among nurses, it seems necessary to hold training courses on dealing with job risks, minimizing the stress of the work environment, and employing less experienced nurses alongside experienced nurses.

## Data Availability

Data will be made available on request.

## Abbreviations

CI: Confidence Interval
HIV: Human Immunodeficiency Virus
NSI: Needle Stick Injury
PRISMA: Preferred Reporting Items for Systematic Reviews and Meta-Analyses
PROSPERO: The International Prospective Register of Systematic Reviews
SID: Scientific Information Database
STROBE: STrengthening the Reporting of OBservational Studies in epidemiology
